# A System of Obstetric Medicine and Surgery

**Published:** 1885-12

**Authors:** 


*A System of Obstetric Medicine and Surgery.* By ROBERT BARNES and FANCOURT BARNES. Two Vols. Pp. 592 and 738. London: SMITH, ELDER & Co. 1885.

To seek to estimate this work by any standard lower than the highest, would be unjust to its authors. It is mainly the production of a man widely and honourably known as of high repute in his department. Others have assisted him: his son, chiefly in the sections devoted to operation; Mr. Noble Smith, in the malformations and diseases of the embryo; and Professor Milnes Marshall is entirely responsible for the section on embryology. This subdivision of labour is not without its significance. We do not for a moment question its value; but we confess to a feeling of something akin to trepidation when we face the fact that a teacher and specialist in obstetrics, holding the position of Dr. Barnes, does not feel himself competent to guide us over the whole of his selected field of study and practice. If every future writer is to be as honest as Dr. Barnes in this respect, we fear that handbooks will be displaced by systems, and systems become expanded into encyclopædias. In the meantime, what of the student and our common three score years and ten?

That the work fully and adequately represents the modern science and art of obstetrics goes almost without saying. Little more is left to the reviewer than to try to gauge the degree of its excellence. We confess to being disappointed with the engravings, not with their artistic character, for that is everywhere unimpeachable, but with their selection. Surely the sectional drawing of the female pelvic organs (vol. i., p. 20), after Sappey, has been abundantly proved to be erroneous. And many, too many, of the others have been produced and reproduced almost *ad nauseam* in works which have been before the profession for years. It may be hypercritical to take exception to this hereditary transmission of engravings; but we think a writer with the capacities and opportunities of Robert Barnes ought, like Thackeray, to make an endeavour to illuminate his own work with his own candles. The only other depreciatory remark we have to make refers to the index: it is not bad, but it might easily be better. And we hope the author will see his way in a future edition—which will, doubtless, soon be called for—to provide a general index for the whole work.

Returning to the excellences of the work, we think special commendation is merited for completeness, balance, arrangement, and language. Nothing of importance in obstetrics is omitted; much that receives little or no mention in other works is included; and a just and fair proportion of space, without personal predilection or bias, is given to each subject. Each matter is placed in its proper position, and adequately treated; there is little or no overlapping; all is clear, definite, and straightforward. Of great value are the frequent summaries and conclusions placed, in well formulated sentences, at the end of many of the disquisitions. Most of all would we commend the language. It is no small boon to the jaded intellect, in these bustling times, to be able to travel in easy conveyances over well-paved roads; and we heartily thank Dr. Barnes for the comfort with which he has carried our mind right through to the end. His clean-cut, clear, and elegant sentences are a positive pleasure to read. One who writes so must think so: it is a strong presumption in favour of the excellence of a work that its author knows how to speak out with clearness and definiteness, as well as elegance and vigour. We are willing to believe that our readers will find the presumptive virtues of Dr. Barnes's work more than borne out by its perusal.

